# Synthesis and Spectroscopic and Biological Activities of Zn(II) Porphyrin with Oxygen Donors

**DOI:** 10.1155/2014/782762

**Published:** 2014-03-16

**Authors:** Gauri Devi Bajju, Sujata Kundan, Madhulika Bhagat, Deepmala Gupta, Ashu Kapahi, Geeta Devi

**Affiliations:** ^1^Department of Chemistry, University of Jammu, New Campus, Baba Sahib Ambedkar Road, Jammu, Jammu and Kashmir 180 006, India; ^2^School of Biotechnology, University of Jammu, New Campus, Baba Sahib Ambedkar Road, Jammu, Jammu and Kashmir 180 006, India

## Abstract

Results of investigation of the physicochemical properties of zinc complexes containing substituted phenols as axial ligand having general formula [X-Zn-t(*p*-CH_3_) PP] [where X = different phenolates as axial ligand] in impurity-free organic solvent are presented. The four-coordinated zinc porphyrin accepts one axial ligand in 1 : 1 molar ratio to form five-coordinated complex, which is purified by column chromatography and characterized by physicochemical, biological evaluation and TGA/DTA studies. Absorption spectra show two principal effects: a red shift for phenols bearing substituted electron releasing groups (−CH_3_, −NH_2_) and blue shift for phenols bearing electron withdrawing groups (−NO_2_, −Cl) relative to Zn-t(*p*-CH_3_) PP, respectively. ^1^H NMR spectra show that the protons of the phenol ring axially attached to the central metal ion are merged with the protons of the porphyrin ring. Fluorescence spectra show two fluorescence peaks in the red region with emission ranging from 550 nm to 700 nm. IR spectra confirm the appearance of Zn-N_Por_ and Zn-O vibrational frequencies, respectively. According to the thermal studies, the complexes have a higher thermal stability and the decomposition temperature of these complexes depends on the axial ligation. The respective complexes of X-Zn^II^-t(*p*-CH_3_) PP were found to possess higher antifungal activity (up to 90%) and higher *in vitro* cytotoxicity against human cancer cells lines.

## 1. Introduction

The involvement of porphyrins in many biological processes and the possibility of tailoring their physical and chemical properties at the molecular level make the porphyrins and metalloporphyrins versatile synthetic base material for research areas due to their immense biological and fascinating importance in many technical applications including, but not limited to, sensors, solar cells, as catalysts [1–6], optical monomers [7, 8], photocatalysts [9–12], photosensitizers in photodynamic therapy (PDT) [13], supramolecular chemistry [14, 15], ionophores [16], and for the treatment of tumors and malignant tissues [17] in combination with electromagnetic radiation or radioactive emissions, as photosensitizers for dye sensitized solar cells (DSSCs) [18, 19]. They are also regarded as nature's choice catalysts and carry out a remarkable spectrum of bioenergetics reaction ranging from photosynthetic energy transduction to generation of ammonia, regiospecific oxygen transfer (hydroxylation and epoxidation), and conversion of carbon dioxide to hydrocarbons. The free base 5,10,15,20-*meso*-tetraphenylporphyrin (H_2_TPP) and the* meso*-substituted-tetra(*ortho*- and* para*-phenyl) porphyrin derivatives (–CH_3_, –OCH_3_, –Cl, or –NEt_2_) have been reviewed in literature and were synthesized according to the documented procedure [20]. Over the past decades, many examples of axial coordination properties of metalloporphyrins with S, O, P, and N bases have been reported [21, 22]. The interaction of metalloporphyrins with donor molecules via axial coordination either in their ground and excited state can strongly influence the absorption properties and the efficiency of energy or electron transfer processes [23, 24]. This ability of metalloporphyrins to attach additional ligand (extra coordination) determines their role in enzyme and catalytic processes. Mono- or bidentate complexes form, depending on the system of substituent in the porphyrin macrocycle, central ion, or the nature and concentration of the extra ligand. The reactions of extra coordination attract attention on both theoretical and experimental researches [25]. Also, the detailed studies on the solvation and axial ligation properties of ZnTPP have been reported [26, 27]. These investigations have shed light on how axial ligands induce changes in the spectral absorption features and the electrochemistry of metalloporphyrins. The ability for numerous chemical modifications and the large number of different mechanisms by which porphyrins affect microbial and viral pathogens place porphyrins into a group of compounds with an outstanding potential for discovery of novel agents, procedures, and materials active against pathogenic microorganisms [28]. Metalloporphyrins are the basis of new antifungal, antiparasitic, and anticancer drugs because modification of the porphyrin periphery confers qualitatively a new spectrum of activities to metalloporphyrins [29, 30]. Metal complexes are well known to accelerate drug action and the efficiency of a therapeutic agent can often be enhanced upon coordination with a metal ion [31]. Of particular interest metal ions such as zinc, which is a natural component of insulin required for the regulation of sugar metabolism and it is also incorporated into the catalytic proteins to act as a metalloenzyme that facilitate a multitude of chemical reactions needed for life. Zn(II) metal complexes with different ligands show overall good potential for antibacterial, antifungal, antioxidant, and anticancer activities [32, 33]. Zinc also forms low molecular weight complexes and, therefore, proves to be more beneficial against several diseases. Various biological aspects of the metal-based drugs/ligands entirely depend on the ease of cleaving the bond between the metal ion and the ligand. As a consequence, it is essential to understand the relationship between ligand and the metal in biological systems. With this objective, we aimed at synthesis, spectroscopic characterization, and biological studies of axial zinc(II)-5,10,15,20-*meso-*tetra(*para*-methylphenyl)porphyrins with different phenols as axial ligand.

## 2. Experimental

### 2.1. Materials and Instruments

All the chemicals were of analytical grade and used as received unless otherwise noted. Pyrrole (Fluka, Switzerland) was distilled at room temperature over potassium hydroxide (KOH) pellets under reduced pressure before use.* p-Tolualdehyde* (*p*-methylbenzaldehyde) (Aldrich, USA), propionic acid (Qualigens, India), silica gel (60–120 mesh) and silica gel (TLC grade, particle size = 75 *μ* (Merck, Germany), aluminum oxide (basic) for column chromatography (Fluka, Switzerland), and zinc acetate (Zn(OAc)_2_ ·2H_2_O) (E. Merck, India) were used as supplied. Organic solvents were degassed by purging with prepurified nitrogen gas and dried before use. The various phenols used were of AR grade (Sisco Research Laboratories Pvt. Ltd.) and used without further purification.

The optical absorption spectra of the compounds were recorded on a Hitachi U-3400, Lambda 35 UV-Vis spectrophotometer and Elico spectral treats UV-Vis spectrometer using a pair of matched quartz cells of 10 mm path length at an ambient temperature. The oscillator strength (*f*) of the transitions in absorption spectra was calculated from the expression [34]

(1) $$\begin{array}{c} f=4.33\times 10^{-9}\varepsilon \Delta \nu_{1/2}, \end{array}$$

where *ε* is the molar absorption coefficient in dm^3^ mol^−1^ cm^−1^ and Δ*ν*_1/2_ is the full width at half maximum in cm^−1^. IR spectra of complexes over the region 4000–400 cm^−1^ were recorded on PERKIN ELMER 580 B spectrophotometer at room temperature using KBr discs or Nujol mulls which confirms the (M-N) and (M-L) vibration [35, 36]. The ^1^H NMR spectra were recorded on a Bruker Avance II 400 (MHz) NMR spectrometer in CDCl_3_ using tetramethylsilane (TMS) as internal standard. Porphyrin solutions (0.5 mL) of 10^−2^ to 10^−3^ m in CDCl_3_ were used for ^1^H NMR studies. Carbon, hydrogen, and nitrogen were analyzed microanalytically using CHNS analyzer Leco model 932, USA, at a temperature of about 1000°C using helium as carrier gas and oxygen for combustion. The MALDI mass spectra were recorded in the electron-impact mode on a Finnigan 3300 spectrometer using chloroform or methanol as solvent. The steady state fluorescence measurements were performed on synergy MX Biotek Multimode reader using a quartz cell of 1 cm path length at ambient temperature. The thermogravimetric analyses (TGA) and differential thermal analyses (DTA) were performed on a Linseis STA PT-1000 in air atmosphere at a heating rate of 10°C/min. The right angle detection was employed for monitoring the fluorescence.

### 2.2. Biological Studies

#### 2.2.1. Antifungal Studies

The* in vitro* antifungal activity has been done by disc diffusion method (DDM) against the pathogen and* in vitro* cytotoxicity against human cancer cell lines.* In vitro* antifungal activity of some of the investigated compounds was tested by agar plate technique against the pathogen “*Sclerotium rolfsi*” by the poisoned food method using potato dextrose agar (PDA) (glucose 20 g, starch 20 g, and agar-agar 20 g in 1000 mL distilled water) nutrient as the medium. Solution of the test compounds in DMSO (100 ppm, 200 ppm, and 300 ppm concentrations) was prepared and mixed with the PDA. The medium was then poured into sterilized Petri plates and the spores of fungi were placed on the medium with the help of inoculum's needle inside laminar flow. The plates were inoculated with seven-day-old culture of the pathogen by placing 2 mm bit of the compound under investigation with different concentration in the centre of plates. The inoculated plates were incubated at 27°C for 5 days. The linear growth of fungus in control and treatment was recorded at different concentrations of the complexes after 5 days. The growth of  “*Sclerotium rolfsi*” over control was calculated as per Vincent [37]:

(2) $$\begin{array}{c} \%\;\;\text{inhibition}\;\;\left(I\right)=\frac{\left(C-T\right)}{C}\times 100, \end{array}$$

where *I* = percent inhibition, *C* = mean growth of fungus in (mm) in control, and *T* = mean growth of fungus in (mm) in treatment.

#### 2.2.2. *In Vitro* Cytotoxicity against Human Cancer Cell Lines

*Cell Lines and Cell Cultures.* The human cancer lines were obtained either from National Center for Cell Science, Pune, India, or IIIM, Jammu, J&K, India. The human prostate (PC-3), lung (A-549), and acute lymphoblastic leukemia (THP-1) cell line was grown and maintained in RPMI-1640 medium, pH 7.4, whereas DMEM was used for Breast (MCF-7). The media were supplemented with FCS (10%), penicillin (100 units/mL), streptomycin (100 µg/mL), and glutamine (2 mM) and cells were grown in CO_2_ incubator (Heraeus, GmbH, Germany) at 37°C with 90% humidity and 5% CO_2_. Cells were treated with samples dissolved in DMSO while the untreated control cultures received only the vehicle (DMSO, <0.2%).

*Cytotoxicity Assay. In vitro* cytotoxicity against human cancer cell lines was determined using sulphorhodamine B dye assay [38, 39]. Both test samples stock solutions were prepared in DMSO and serially diluted with growth medium to obtain desired concentrations.

### 2.3. Synthesis of Axially Ligated Zn(II) Porphyrins Complexes

#### 2.3.1. Synthesis of 5,10,15,20-*Meso*-tetra(*p*-methylphenyl)porphyrin [H_2_t(*p*-CH_3_)PP]

**Scheme 1:**
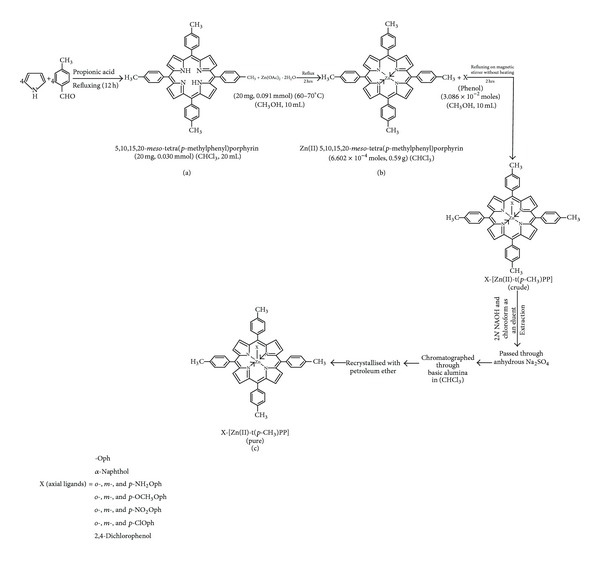
General synthetic route for the synthesis of zinc(II)-5,10,15,20-*meso*-tetra(*para*-methylphenyl)porphyrin containing different phenols as axial ligand.

The preparation of H_2_-t(*p*-CH_3_)PP was carried out by condensation of pyrrole with* p*-tolualdehyde in refluxing propionic acid, H_2_-t(*p*-CH_3_)PP purified by column chromatography using chloroform as eluent (Scheme 1(a)). A second moving band was collected after evaporation of the solvent furnished violet colour as a title compound. UV-Visible (*λ*_nm_(nm)(CHCl_3_)):430, 516, 553, 592, 649; ^1^H NMR (CDCl_3_): −2.77(s) (Imino-H), 8.86 (s,8H,*β*_py_-H),* meso*-aryl protons 8.11(d,8H,H_*o*_), 7.56 (d,8H,H_*m*_), 2.64 (s, 12H,H_me_); IR spectra (in KBr) (cm^−1^): imino *ν*(N–H) at 3446, aromatic *ν*(C–H) at 2964, *ν*(C–N) at 1350, *ν*(C=C) at 1650, *ν*(C=N) at 1589, *ν*(CH_3_) at 2855; Anal.Calcd. for C_48_H_38_N_4_(%): C, 86.04; H, 5.71; N, 8.6. Found: C, 86.11; H, 5.89; N, 8.42.

#### 2.3.2. Synthesis of Zinc(II)5,10,15,20-*Meso*-tetra(*p*-methylphenyl)porphyrin [Zn^II^-t(*p*-CH_3_)PP]

H_2_-t(*p*-CH_3_)PP (20 mg, 0.030 mmol) in chloroform (20 mL) and Zn(OAc)_2_·2H_2_O (20 mg, 0.091 mmol) in methanol (10 mL) were refluxed for 2 hrs at 60–70°C till the colour of the solution changed from purple to red. After cooling to room temperature the solvent was removed under reduced pressure and the solid residue was repeatedly washed with water (3 × 60 mL) to remove the excess of zinc acetate. The filtered product was dried over anhydrous sodium sulphate and purified by column chromatography using (Al_2_O_3_) as stationary phase and CHCl_3_ as eluent. Yield of the complex [Zn^II^-t(*p*-CH_3_)PP] (18 mg, 80%) (Scheme 1(b)).

UV-Vis (*λ*_nm_(nm)(CHCl_3_)): 432, 564.2, 609.3; ^1^H NMR (CDCl_3_): 8.65 (s,8H, *β*_py_-H), 7.75 (d,8H,H_*m*_), 8.06 (d,8H,H_*o*_), 2.69 (s,12H,–CH_3_); IR spectra (inKBr) (cm^−1^): aromatic *ν*(C–H) appears at 2963, *ν*(C–N) at 1349, *ν*(C=C) at 1658, *ν*(C=N) at 1594, *ν*(CH_3_) at 2849, *ν*(Zn–N) at 482; Anal.Calcd. for C_48_H_36_N_4_Zn_1_(%): C, 78.39; H, 4.93; N, 7.61. Found: C, 78.46; H, 4.99; N, 7.72.

#### 2.3.3. Synthesis of 5,10,15,20-*Meso*-tetra(*p*-methylphenyl)porphinatozinc(II)-phenoxide [X-Zn^II^-t(*p*-CH_3_)PP]

It is as follows:

(3) $$\begin{array}{c} \text{Zn}\left(\text{II}\right)\text{-t}\left(p\text{-}{\text{CH}}_{3}\right)\text{PP}+\text{X}⇆\text{X-Zn}\left(\text{II}\right)\text{-t}\left(p\text{-}{\text{CH}}_{3}\right)\text{PP} \end{array}$$

Phenol (X) (3.086 × 10^−2^ moles) in methanol (10 mL) and Zn-t(*p*-CH_3_)PP (6.602 × 10^−4^ moles; 0.59 g) in chloroform (15 mL) were stirred without heating for two hours. After completion of reaction as indicated by TLC, the reaction mixture was extracted with 2*N* NaOH solution and chloroform as an eluent. The compound recovered after extraction was passed through anhydrous Na_2_SO_4_. The solvent was recovered under reduced pressure and chromatographed through basic alumina using chloroform as an eluent, recrystallised with petroleum ether, and characterized by UV-Vis and ^1^H NMR spectra (Scheme 1(c)).

## 3. Results and Discussion

### 3.1. Synthesis and Characterization

The physical measurements and analytical data of all complexes with general formula [X-Zn^II^-(*p*-CH_3_)PP] (where X = different phenolates as axial ligand) are shown in Tables 1–6. All the complexes are coloured, photosensitive to light, and soluble in polar solvents but water insoluble. The data showing growth inhibition of the fungus is given in Table 7.

#### 3.1.1. ^1^H NMR Spectroscopy

**Figure 1:**
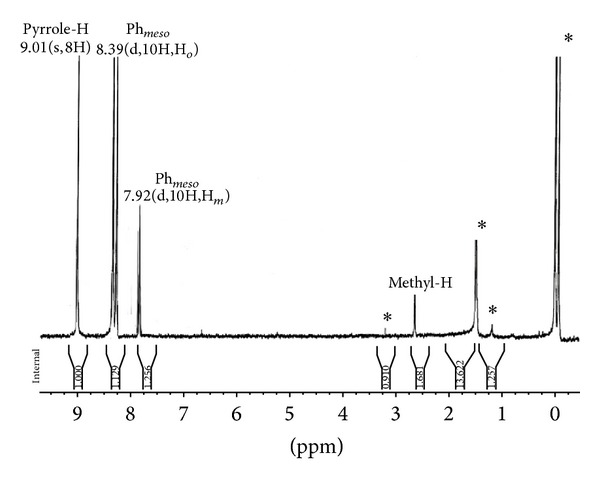
^1^H NMR spectra of* p*-NO_2_phO-Zn-t-(*p*-CH_3_)PP in CDCl3 at 298 K. Starred peaks are solvents impurities.

**Figure 2:**
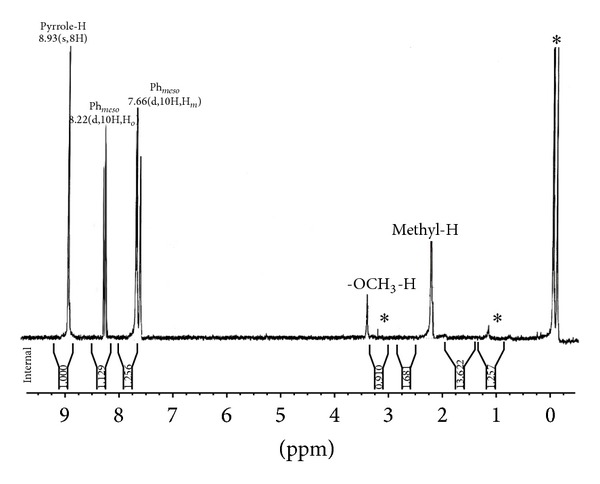
^1^H NMR spectra of* p*-OCH_3_phO-Zn-t-(*p*-CH_3_)PP in CDCl_3_ at 298 K. Starred peaks are solvents impurities.

The ^1^H NMR spectrum of the* meso*-tetra(*p*-methylphenyl)porphyrin and its Zn(II) derivatives containing different phenols as axial ligand was recorded in deuterated chloroform at 298 K (Table 1). The spectrum show a singlet at −2.79 ppm for inner imino protons of the H_2_TPP, while those of H_2_-t(*p*-CH_3_)PP resonate at −2.77 ppm. The* meso*-aryl protons of H_2_TPP resonate as a singlet at 8.19 ppm for* ortho* and 7.59 ppm for* meta* and* para* protons, respectively, but in case of H_2_-t(*p*-CH_3_)PP, the resonance occurs at 8.11 ppm for* ortho* and 7.56 ppm for* meta* protons; that is, resonance is shifted upfield relative to H_2_TPP. The methyl protons of the substituted –CH_3_ group at the* para*-position of the* meso*-aryl ring resonate as a singlet at 2.64 ppm. This effect of* meso*-substitution on the *β*-pyrrole protons and* meso*-aryl protons has earlier been reviewed in literature [40]. Further, in axially ligated zinc(II) complexes of H_2_-t(*p*-CH_3_)PP, a slight difference in the proton resonance is observed depending upon the nature of the axial ligand coordinated via zinc atom. In the case of* p*-NO_2_phO-Zn-t(*p*-CH_3_)PP, Figure 1 indicates that the *β*-pyrrole protons resonate as a singlet at 9.01 ppm and the* meso*-aryl* ortho* protons resonate as doublet at 8.39 ppm and 7.92 ppm for* meso*-aryl* meta* and* para* protons, respectively, which are slightly downfield (deshielded) compared to Zn-t(*p*-CH_3_)PP as well as for H_2_-t(*p*-CH_3_)PP. The methyl protons of the* meso*-aryl rings resonate at 2.65 ppm. But in case of* p*-OCH_3_phO-Zn-t(*p*-CH_3_)PP (Figure 2), the *β*-pyrrole protons resonate as a singlet at 8.93 ppm and the* meso*-aryl* ortho* protons resonate as doublet at 8.22 ppm and 7.66 ppm for* meta* and* para*, respectively, which are slightly upfield (shielded) compared to Zn-t(*p*-CH_3_)PP as well as for H_2_-t(*p*-CH_3_)PP. The methyl protons of the* meso*-aryl rings resonate at 2.31 ppm and the methoxy protons of* para*-methoxy phenolate resonate as singlet at 3.36 ppm. The ^1^H NMR data of various axially ligated Zn(II) complexes of H_2_t(*p*-CH_3_)PP revealed that phenols with electron-withdrawing groups like –NO_2_, –Cl caused slight downfield shift (deshielding) and those with electron releasing group like –CH_3_, –OCH_3_, and –NH_2_ caused upfield shift (shielding) of protons with respect to Zn-t(*p*-CH_3_)PP and H_2_t(*p*-CH_3_)PP complexes.

#### 3.1.2. Absorption Spectroscopy

**Figure 3:**
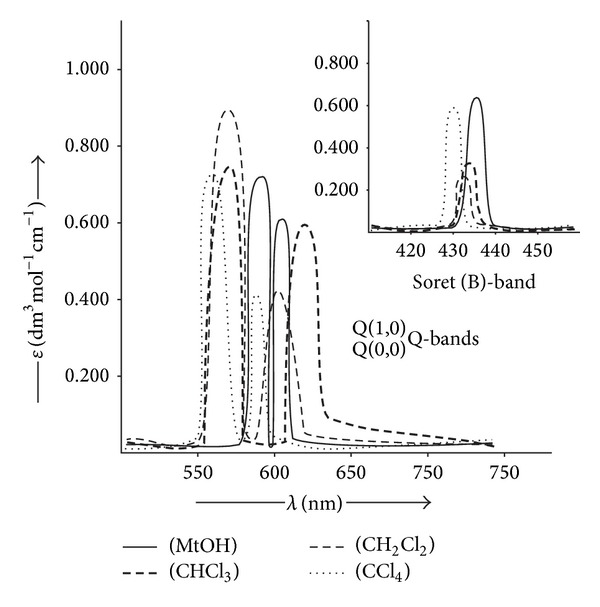
UV-Visible spectra of* p*-NH_2_phO-Zn-t(*p*-CH_3_)PP in different solvents.

The electronic spectra of a typical porphyrin contain one intense band in the near-ultraviolet region of the spectrum around 400 nm (the soret band or B-band) depending on whether the porphyrin is *β*- or* meso*-substituted with *ε* > 2 × 10^5^, followed by four low-intensity absorption bands at 514 nm, 550 nm, 591 nm, and 647 nm (the Q-band), that is, Q_y_(1,0), Q_y_(0,0), Q_x_(1,0), and Q_x_(0,0), respectively. The B- and Q-bands both arise from *π*→π* transition and can be explained by four frontier orbitals (HOMO and LUMO orbitals) (the Gouterman four orbital model). Ongoing from porphyrin to metalloporphyrin, the ring symmetry of the planar macrocycle fragment increases to D_4h_ from D_2h_ due to which the spectrum is simplified. The optical absorption data Zn(II)-5,10,15,20-*meso*-tetra(*p*-methylphenyl)porphyrin containing different phenols as axial ligand in chloroform is listed in (Table 2). The optical absorption spectra of X-Zn-t(*p*-CH_3_)PP (X = different phenols as axial ligand) in chloroform revealed that phenols containing electron withdrawing groups show blue shift (hypsochromic shift) while those having electron releasing groups show red shift (bathochromic shift) that is towards longer wavelength. When absorption spectra of axially ligated Zn-t(*p*-CH_3_)PP is recorded in different solvents (Table 3), it was observed that the spectra of* p*-NH_2_phO-Zn-t(*p*-CH_3_)PP (Figure 3) shows only a marginal change in *λ*_max⁡_, absorption coefficient (*ε*), and oscillator strength (*f*) values. The data also reveal that a change in polarity of the solvents does not significantly alter the position of the transition but there is a significant increase in “Fwhm” (*ν*_1/2_) and “*f*” values of transition by increasing the polarity of the solvent. In polar solvents such as methanol, ethanol, CH_2_Cl_2_, CHCl_3_, the *π* → π* band undergoes red shift and was stable but in nonpolar solvents such as benzene, toluene, and CCl_4_; however, the complexes usually displayed a spectral drift for a period of time. It is observed that, for all the axially ligated Zn(II) derivatives, B- and Q-bands exhibit a red shift on increasing the polarity of the solvents in the order MtOH > CHCl_3_ > CH_2_Cl_2_ > CCl_4_. As in the case of* p*-NH_2_phO-Zn-t(*p*-CH_3_)PP (Figure 3), *λ*_max⁡_ values in MtOH were observed at 435.9 nm, 589 nm, and 604 nm while in CHCl_3_, CH_2_Cl_2_, and CCl_4_ were observed at 433.9 nm, 570.9 nm, and 604.6 nm; 432.8 nm, 568.4 nm, and 601 nm; and 430 nm, 560.1 nm, and 586 nm, respectively. However, both B(0,0) and Q(1,0) exhibit only a small change in the* f* value, which depend on the nature of the solvent. The* f* value for Q(1,0) in MtOH, CHCl_3_, CH_2_Cl_2_, and CCl_4_ was observed at 0.206755, 0.253434, 0.1878264, and 0.1331380, respectively. It was found that, with the increase in polarity of the solvents, the axially ligated Zn(II) metalloporphyrin with different phenols as axial ligand shows the progressive broadening of the B- and Q-bands indicating that the magnitude of change of the “*f*” value depends on the nature of the solvent and also reveals the relative strength of *π* → π* interactions.

#### 3.1.3. Infrared Spectroscopy

The vibrational spectroscopy (IR spectroscopy) can provide ample information about the structure of porphyrin and metalloporphyrin. The IR spectra of free-base porphyrin, H_2_-t(*p*-CH_3_)PP, and its axially ligated zinc(II) metal derivatives containing different phenols as axial ligand exhibit strong absorption band at 2855 cm^−1^ (2850–3000 cm^−1^) due to –CH_3_ group at* meso*-phenyl position. The metallation of porphyrin and axial ligation with different phenols were further supported by the emergence of two new bands at 500–400 cm^−1^ and 650–350 cm^−1^ assigned to zinc-nitrogen (Zn-N_Por_) [41] and zinc-oxygen (Zn–O) vibrational modes, respectively. In order to determine the mode of bonding of different phenols as ligand with zinc, the IR spectra of ligands were compared with those of corresponding complexes (Table 4). The IR spectra of free-base porphyrin (H_2_-t(*p*-CH_3_)PP) and its axially ligated zinc(II) metal derivatives containing different phenols as axial ligand showing different band frequencies agree well with the literature [42]. For example, in the IR spectra of 2,4-Cl_2_phO-Zn-t(*p*-CH_3_)PP, aromatic *ν*(C–H) vibrates at 2963 cm^−1^, *ν*(C–N) at 1349 cm^−1^, *ν*(C=C) at 1659 cm^−1^, *ν*(C=N) at 1594 cm^−1^, *ν*(CH_3_) at 2846 cm^−1^, and *ν*(Zn-N_Por_) at 476 cm^−1^ and *ν*(Zn–O) of phenolate appears at 517 cm^−1^ and in* p*-OCH_3_phO-Zn-t(*p*-CH_3_)PP, aromatic *ν*(C–H) vibrates at 2964 cm^−1^, *ν*(C–N) at 1350 cm^−1^, *ν*(C=C) at 1654 cm^−1^, *ν*(C=N) at 1590 cm^−1^, *ν*(CH_3_) at 2859 cm^−1^, *ν*(Zn-N_Por_) at 481 cm^−1^, *ν*(Zn–O) of phenolate at 526 cm^−1^ and the methoxy (–OCH_3_) group at the* para*-position of the phenolate vibrates at 2850 cm^−1^ for (*ν*_1_)(C–H), 1020 cm^−1^ for (*ν*_2_)(C–O–C)_sym_, and 1261 cm^−1^ for (*ν*_3_)(C–O–C)_asym_, respectively. The formations of the axially ligated Zn(II), metal complexes were also confirmed by their mass spectral data given in the experimental section.

#### 3.1.4. Mass Spectroscopy and Elemental Analysis

The mass spectra of several porphyrins and their metalloderivatives have been obtained by mass spectroscopy technique. The molecular mass [43] spectra of porphyrins and their derivatives are best recorded at the lowest possible temperature (usually approx. 200–250°C). The intensity of molecular ion in the mass spectra of porphyrin has also been used in the study of deuteration (as a general example of electrophilic substitution) of porphyrins, metalloporphyrins, and some reduced derivatives.  Table 5 summarizes the mass spectra and elemental analysis data of axially ligated Zn(II) derivates.

#### 3.1.5. Fluorescence Spectroscopy

**Figure 4:**
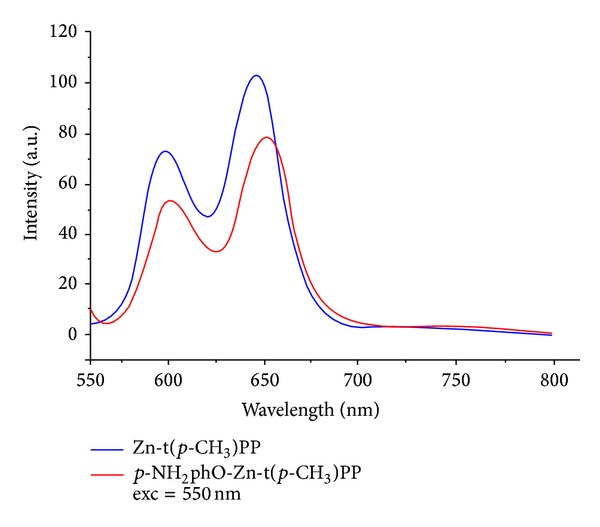
Fluorescence spectral data of Zn-t(*p*-CH_3_)PP and* p*-NH_2_phO-Zn-t(*p*-CH_3_)PP in methanol at excitation 550 nm.

An important and unique feature of porphyrins and their metal derivatives is their emission spectra. The fluorescence emission data [44, 45] of the porphyrins provide important information on the singlet excited state properties. The optical properties are affected by the presence of substituents at *β*-pyrrole and at* meso*-aryl positions of the tetraphenylporphyrins. The axially ligated metalloporphyrins exhibited two fluorescence bands, one from S_2_ → S_0_ (B-band) and the other from S_1_ → S_0_ (Q-band). Internal conversion from S_2_ to S_1_ is rapid so that there is hardly any fluorescence absorption detected from S_2_ → S_1_. The S_2_ → S_0_ (soret band) fluorescence is about two orders of magnitude weaker than S_1_ → S_0_ of Q-band emission. However, the emission bands of axially ligated Zn(II) porphyrins are red shifted compared to Zn-t(*p*-CH_3_)PP. The intensities of low energy Q(1,0) are more intense than high energy Q(0,0) band in contrast to that observed for the free-base porphyrins. On comparing the fluorescence behavior of Zn-t(*p*-CH_3_)PP with* p*-NH_2_phO-Zn-t(*p*-CH_3_)PP in dry methanol at room temperature using excitation at ~550 nm (Table 6) (Figure 4), it is clear from the figure that the emission bands of* p*-NH_2_phO-Zn-t(*p*-CH_3_)PP are red shifted compared to Zn-t(*p*-CH_3_)PP which is due to the electron donating effect of the amino (–NH_2_) group attached to the phenolate ion. This fluorescence analysis procedure is of great importance in identifying the porphyrin chemosensors for selective detection of amine compounds of biological and technical interest. In addition, the possibilities of producing solid-state solar cells by synthesis of semiconductors with porphyrin compounds have been intensely explored.

#### 3.1.6. TGA/DTG Studies

**Figure 5:**
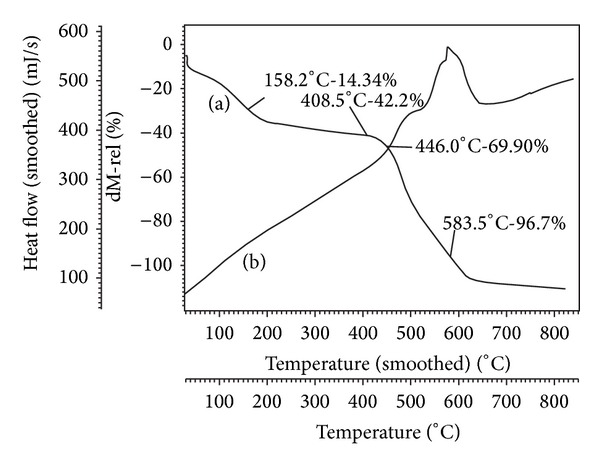
TG curve (a) and DTA curve (b) of* p*-OCH_3_phO-Zn-t(*p*-CH_3_)PP.

*Thermal Analysis of p-OCH*_*3*_ *phO-Zn-t-(p-CH*_*3*_ *)PP.* Thermogravimetric analyses were performed in an air atmosphere at a heating rate of 10°C/min to examine thermal stability of the compound. The TG curve of the complex* p*-OCH_3_phO-Zn-t-(*p*-CH_3_)PP (Figure 5) shows a continuous weight loss starting from 150°C to 800°C, when a stable oxide of ZnO is formed. The TG curve shows an initial weight loss of about 14.34% (the theoretical value = 14.7%) observed between 140°C and 170°C and is attributed to the removal of* para*-methoxy-phenyl ring as axial group. In the range of 200°C to 425°C, up to 42.2% of the mass had been lost due to the loss of tetraphenyl group (the theoretical value = 42.39%). At 446.0°C, up to 69.90% (the theoretical value = 70.79%) of the total mass had been lost, corresponding to the collapse of macrocyclic ligand. The organic moiety decomposes further with increasing temperature. Further in the range of 450°C–600°C, the weight loss reaches up to 96.7% which is attributed to the removal of pyrrole groups and complete decomposition of macrocyclic ligand and finally ZnO is remained (the theoretical value = 96.9%).

Simultaneously, there were three exothermal peaks at 492°C, 563°C, and 580°C on the DTA curve, corresponding to the major weight loss in the ligand H_2_-t(*p*-CH_3_)PP (460°C–580°C). The small exothermic peak corresponds to the decomposition of the ligand and the loss of chains of the porphyrin ring and the large exothermic peak corresponds to the collapse of the porphyrin skeleton.

#### 3.1.7. Biological Evaluation

**Figure 6:**
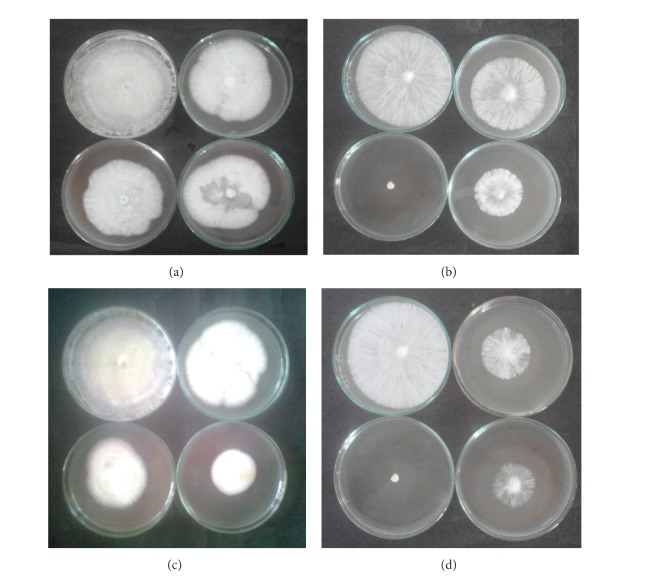
(a) Antifungal activity of* p*-NH_2_phO-Zn-t(*p*-CH_3_)PP, (b) antifungal activity of* p*-OCH_3_phO-Zn-t(*p*-CH_3_)PP, (c) antifungal activity of* p*-NO_2_phO-Zn-t(*p*-CH_3_)PP, and (d) antifungal activity of* p*-ClphO-Zn-t(*p*-CH_3_)PP.

*Antifungal Activity.* Antifungal activities of some complexes were studied against one fungal strain “*Sclerotium rolfsi*.” It is concluded that all the synthesized complexes showed overall good activity against this antifungal strain up to 90% (Figures 6(a), 6(b), 6(c), and 6(d)). From the results found (Table 7), it has been concluded that, on increasing the concentration of the complexes, the colony diameter of the fungus decreases and hence percent inhibition increases. On doubling the concentration of the complexes, the percent inhibition also doubles, which shows linear relationship between concentration and percent inhibition. The increase in biological activity is due to faster diffusion of metal complexes as a whole through the cell membrane or due to combined effect of metal atom and the ligand. Such increased activity of the metal complexes can be explained on the basis of Overtone's concept [46] and Tweedy's chelation theory [47]. The lipid membrane that surrounds the cell favors the passage of only lipid soluble materials, due to liphophilicity being an important factor which controls the antimicrobial activity. On chelation, the polarity of the metal ion will be reduced to a greater extent due to overlap of the ligand orbital and partial sharing of the positive charge of the metal ion with donor group.

**Figure 7:**
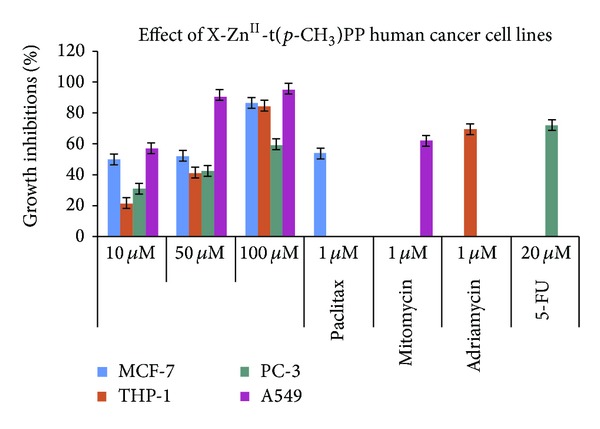
*In vitro* cytotoxicity of* p*-NO_2_phO-Zn-t(*p*-CH_3_)PP complexes against human cancer cell lines.

*In Vitro Cytotoxicity.* Evaluation of* in vitro* cytotoxicity of the corresponding ligand was also observed against four human cancer cell lines, namely, Breast (MCF-7), Leukemia (THP-1), Prostate (PC-3), and Lung (A549), at different concentrations by using SRB assay as shown in Figure 7. Dose dependent percent growth inhibition was observed against all the cancer cell lines. Among the substituted oxygen donor ligands,* p*-NO_2_phO-Zn-t(*p*-CH_3_)PP showed prominent activity against three A549, MCF-7, and THP-1 human cancer cell lines. Highest growth percent inhibition was observed against Lung cancer cell line and lowest percent growth inhibition was observed against Prostate cancer cell line by the ligand. The percent growth inhibition observed for the ligand was 57, 90, and 95 against Lung, 50, 52, and 86 against Breast, and 21, 41, and 84 against leukemia cancer cell lines at 10, 50, and 100 µM, respectively, because the presence of electron-withdrawing nitro group (–NO_2_) on the phenolic ring in general increases the antimicrobial activities of the tested metal complexes compared to complexes having no substituent. These results suggested that metal complexes had effective improvement of bioavailability, and electron-withdrawing nitro group had effective and direct impact on selective anticancer activities. Hence, therefore, the ligand* p*-NO_2_phO, which is axially ligated with Zn-t(*p*-CH_3_)PP, shows overall better activity than its free base H_2_-t(*p*-CH_3_)PP and metallated Zn-t(*p*-CH_3_)PP. The complex* p*-NO_2_phO-Zn-t(*p*-CH_3_)PP showed less than 59% growth inhibition against Prostate (PC-3) human cancer cell line.

## 4. Conclusion

**Figure 8:**
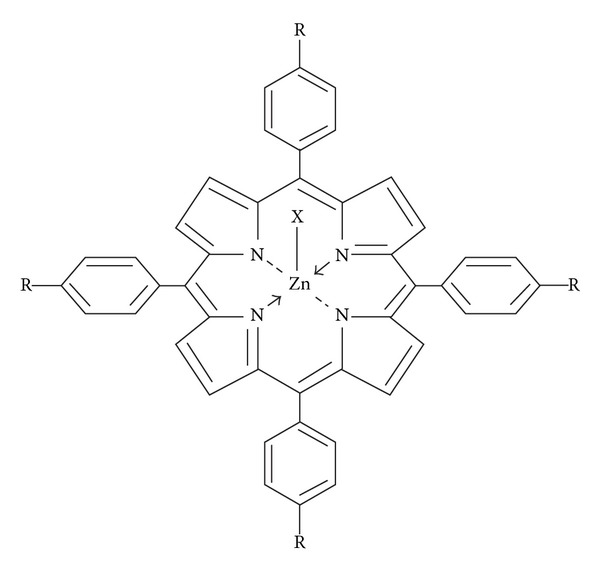
Proposed structure of axially ligated zinc(II) porphyrins.

On the basis of physicochemical and spectral evidences it is found that all the complexes with a general formula X-Zn-t(*p*-CH_3_)PP (X = different phenolates as axial ligand) in which the four-coordinate zinc porphyrin will accept one and only one axial ligand in 1 : 1 molar ratio to form five-coordinated complexes. The proposed structure for the complexes under investigation with general formula [X-Zn-t(*p*-CH_3_)PP] is given as Figure 8. Also, biological evaluation (antifungal and anticancer activities) of the synthesized complexes shows that these complexes have potential against fungal growth. Moreover, for anticancer activity, highest growth percent inhibition was observed against Lung cancer cell line and lowest percent growth inhibition was observed against Prostate cancer cell line by the ligand.

## Figures and Tables

**Table 1:** ^
1^H NMR data^a^ of free base H_2_-t(*p*-CH_3_)PP and axially ligated X-Zn-t(*p*-CH_3_)PP (X = different phenols as axial ligand) in CDCl_3_ at 298 K.

Porphyrins	*β*-Pyrrole protons	Imino protons	*Meso*-aryl protons	Other protons
H_2_t(*p*-CH_3_)PP [(C_48_H_38_N_4_)]	8.86 (s)	−2.77 (s)	8.11(d, 8H, H_*o*_) 7.56(d, 8H, H_*m*_)	2.64(s, 12H, H_me_)
Zn-t(*p*-CH_3_)PP [(C_48_H_36_N_4_Zn_1_)]	8.65 (s)	—	8.06(d, 8H, H_*o*_) 7.75(m, 8H, H_*m*_)	2.69(s, 12H, H_me_)
phO-Zn-t(*p*-CH_3_)PP [(C_6_H_5_O)Zn(C_48_H_36_N_4_)]	8.99 (s)	—	8.41(d, 10H, H_*o*_) 7.76(d, 11H, H_*m*,*p*_)	2.71(s, 12H, H_me_)
*p*-Cl-Zn-t(*p*-CH_3_)PP [(C_6_H_4_ClO)Zn(C_48_H_36_N_4_)]	9.2 (s)	—	8.29(d, 10H, H_*o*_) 7.92(d, 10H, H_*m*_)	2.9(s, 12H, H_me_)
*p*-OCH_3_phO-Zn-t(*p*-CH_3_)PP [(C_7_H_7_O_2_)Zn(C_48_H_36_N_4_)]	8.93 (s)	—	8.22(d, 10H, H_*o*_) 7.66(d, 10H, H_*m*_)	2.31(s, 12H, H_me_) 3.36(s, 3H, H_ome_)
*p*-NH_2_phO-Zn-t(*p*-CH_3_)PP [(C_6_H_6_NO)Zn(C_48_H_36_N_4_)]	8.90 (s)	—	8.26(d, 10H, H_*o*_) 7.69(m, 10H, H_*m*_)	2.36(s, 12H, H_me_) 5.09(s, 2H, H_NH_2__)
2,4-Cl_2_phO-Zn-t(*p*-CH_3_)PP [(C_6_H_3_Cl_2_O)Zn(C_48_H_36_N_4_)]	9.4 (s)	—	8.33(d, 10H, H_*o*_) 7.96(d, 10H, H_*m*_)	3.3(s, 12H, H_me_)
*p*-NO_2_phO-Zn-t(*p*-CH_3_)PP [(C_6_H_4_NO_3_)Zn(C_48_H_36_N_4_)]	9.01 (s)	—	8.39(d, 10H, H_*o*_) 7.92(d, 10H, H_*m*_)	2.65(s, 12H, H_me_)

^a^ *δ* in ppm; the nature of splitting pattern(s): (s = singlet, d = doublet, t = triplet, and m = multiplet); number of proton(s) and their location in the porphyrins, respectively, are given in parenthesis; *o* = ortho; *p* = para; *m* = meta.

**Table 2:** Optical absorption data of X-Zn-t(*p*-CH_3_)PP (X = different phenols as axial ligand) in CHCl_3_ showing *λ*_max⁡_ together with log *ε* and *ν*_1/2_.

Porphyrins	B-bands *λ*_max⁡_, (log *ε*), *ν*_1/2_ (nm), (dm^3^mol^−1^cm^−1^), (cm^−1^)	Q-bands *λ*_max⁡_, (log *ε*), *ν*_1/2_ (nm), (dm^3^mol^−1^cm^−1^), (cm^−1^)
H_2_t(*p*-CH_3_)PP [(C_48_H_38_N_4_)]	430, (5.986), 989.4	516, 553, 592, 649
Zn-t(*p*-CH_3_)PP [(C_48_H_36_N_4_Zn_1_)]	432, (5.824), (995.3)	564.2, (4.218), 789.3; 609.3, (4.160)
phO-Zn-t(*p*-CH_3_)PP [(C_6_H_5_O)Zn(C_48_H_36_N_4_)]	430.2, (5.771), (997.9)	563.8, (4.312), 785.1; 598.6, (4.289)
*α*-Naphthol-Zn-t(*p*-CH_3_)PP [(C_10_H_7_O)Zn(C_48_H_36_N_4_)]	431.4, (5.798), (1008)	564.0, (4.418), 829.4; 600.4, (4.389)
*o*-OCH_3_phO-Zn-t(*p*-CH_3_)PP [(C_7_H_7_O_2_)Zn(C_48_H_36_N_4_)]	432, (5.833), (980.8)	569.4, (4.484), 826.2; 609.6, (4.338)
*m*-OCH_3_ph-Zn-t(*p*-CH_3_)PP [(C_7_H_7_O_2_)Zn(C_48_H_36_N_4_)]	431.9, (5.845), (978.9)	570.2, (4.521), 789.3; 608.3, (4.432)
*p*-OCH_3_phO-Zn-t(*p*-CH_3_)PP [(C_7_H_7_O_2_)Zn(C_48_H_36_N_4_)]	432.4, (5.808), (984.1)	567, (4.448), 822.4; 604, (4.392)
*o*-NH_2_phO-Zn-t(*p*-CH_3_)PP [(C_6_H_6_NO)Zn(C_48_H_36_N_4_)]	433.4, (5.806), (998)	572.4, (4.527), 796.2; 606.3, (4.486)
*m*-NH_2_phO-Zn-t(*p*-CH_3_)PP [(C_6_H_6_NO)Zn(C_48_H_36_N_4_)]	433.2, (5.696), (992.4)	570.4, (4.456), 698.6; 605.1, (4.412)
*p*-NH_2_phO-Zn-t(*p*-CH_3_)PP [(C_6_H_6_NO)Zn(C_48_H_36_N_4_)]	433.9, (5.859), (986.6)	570.9, (4.432), 699.4; 604.6, (4.398)
*o*-CH_3_phO-Zn-t(*p*-CH_3_)PP [(C_7_H_7_O)Zn(C_48_H_36_N_4_)]	431, (5.964), (987.1)	565, (4.643), 762.1; 596, (4.431)
*m*-CH_3_phO-Zn-t(*p*-CH_3_)PP [(C_7_H_7_O)Zn(C_48_H_36_N_4_)]	431.8, (5.839), (986.4)	564, (4.682), 760.9; 588, (4.432)
*p*-CH_3_phO-Zn-t(*p*-CH_3_)PP [(C_7_H_7_O)Zn(C_48_H_36_N_4_)]	431.6, (5.969), (986.9)	564, (4.861), 760.6; 587, (4.743)
*o*-NO_2_phO-Zn-t(*p*-CH_3_)PP [(C_6_H_4_NO_3_)Zn(C_48_H_36_N_4_)]	428, (5.942), (998.9)	546, (4.549), 854.2; 581, (4.431)
*m*-NO_2_phO-Zn-t(*p*-CH_3_)PP [(C_6_H_4_NO_3_)Zn(C_48_H_36_N_4_)]	427, (5.841), (995.2)	546, (4.643), 846.9; 580, (4.428)
*p*-NO_2_phO-Zn-t(*p*-CH_3_)PP [(C_6_H_4_NO_3_)Zn(C_48_H_36_N_4_)]	427, (5.872), (995.6)	546, (4.516), 842.8; 578, (4.314)
*o*-ClphO-Zn-t(*p*-CH_3_)PP [(C_6_H_4_ClO)Zn(C_48_H_36_N_4_)]	426, (5.646), (1014.4)	547, (4.569), 872.3; 575, (4.321)
*m*-ClphO-Zn-t(*p*-CH_3_)PP [(C_6_H_4_ClO)Zn(C_48_H_36_N_4_)]	425, (5.781), (992.5)	548, (4.439), 781.9; 579, (4.532)
*p*-ClphO-Zn-t(*p*-CH_3_)PP [(C_6_H_4_ClO)Zn(C_48_H_36_N_4_)]	425, (5.841), (998.6)	548, (4.598), 785.2; 579, (4.514)

**Table 3:** Optical absorption data of X-Zn-t(*p*-CH_3_)PP (X = different phenols as axial ligand) recorded in different solvents with calculated “*f*” values.

Porphyrins	Solvent	B-band *λ*_max⁡_, (log *ε*), *ν*_1/2_ (nm), (dm^3^mol^−1^cm^−1^), (cm^−1^)	Q-bands *λ*_max⁡_, (log *ε*), *ν*_1/2_ (nm), (dm^3^mol^−1^cm^−1^), (cm^−1^)		Oscillator strength (*f* = 4.33 × 10^−9^ *ε*Δ*ν*_1/2_)	
			Q (1, 0)	Q (0, 0)	B-band	Q-band Q (1, 0)
*p*-NH_2_phO-Zn-t(*p*-CH_3_)PP [(C_6_H_6_NO)Zn(C_48_H_36_N_4_)]	MtOH	435.9, 714 (5.638), 45489	589, 925 (4.724), 51621	604, 531 (4.604)	0.140635	0.206755
	CHCl_3_	433.9, 437 (5.329), 23458	570.9, 1096 (4.749), 53403	604.6, 532 (4.598)	0.044387	0.253434
	CH_2_Cl_2_	432.8, 542 (5.261), 18609	568.4, 679 (4.896), 63885	601, 740 (4.423)	0.0436727	0.1878264
	CCl_4_	430, 631 (5.594), 42352	560.1, 594 (4.726), 51764	586, 447 (4.412)	0.115715	0.1331380

*m*-ClphO-Zn-t(*p*-CH_3_)PP [(C_6_H_4_ClO)Zn(C_48_H_36_N_4_)]	MtOH	433.4, 615 (5.964), 71749	564, 459 (4.643), 47858	592, 525 (4.428)	0.19106399	0.0951163
	CHCl_3_	425, 750 (5.781), 58129	548, 455 (4.439), 32674	579, 549 (4.532)	0.1807739	0.0643726
	CH_2_Cl_2_	424, 857 (5.536), 39894	546, 426 (4.552), 41085	573, 503 (4.561)	0.14803905	0.0757845
	CCl_4_	422.8, 820 (5.536), 39894	542, 561 (4.742), 55227	568, 511 (4.431)	0.1416476	0.1341536

*m*-NO_2_phOZn-t(*p*-CH_3_)PP [(C_6_H_4_NO_3_)Zn(C_48_H_36_N_4_)]	MtOH	429, 686 (5.389), 29681	584, 485 (4.643), 49061	616.2, 555 (4.222)	0.0881638	0.1030306
	CHCl_3_	427, 770 (5.872), 66534	546, 426 (4.643), 49061	580, 463 (4.428)	0.2218310	0.0904969
	CH_2_Cl_2_	427.8, 607 (5.418), 31893	544.8, 470 (4.549), 41889	577.4, 367 (4.431)	0.0838246	0.0852483
	CCl_4_	424.4, 766 (5.569), 43415	542.9, 540 (4.321), 24493	540.2, 584 (4.249)	0.1439980	0.0572695

**Table 4:** Main vibrational frequencies corresponding to the various groups in X-Zn-t(*p*-CH_3_)PP (X = different phenols as axial ligand).

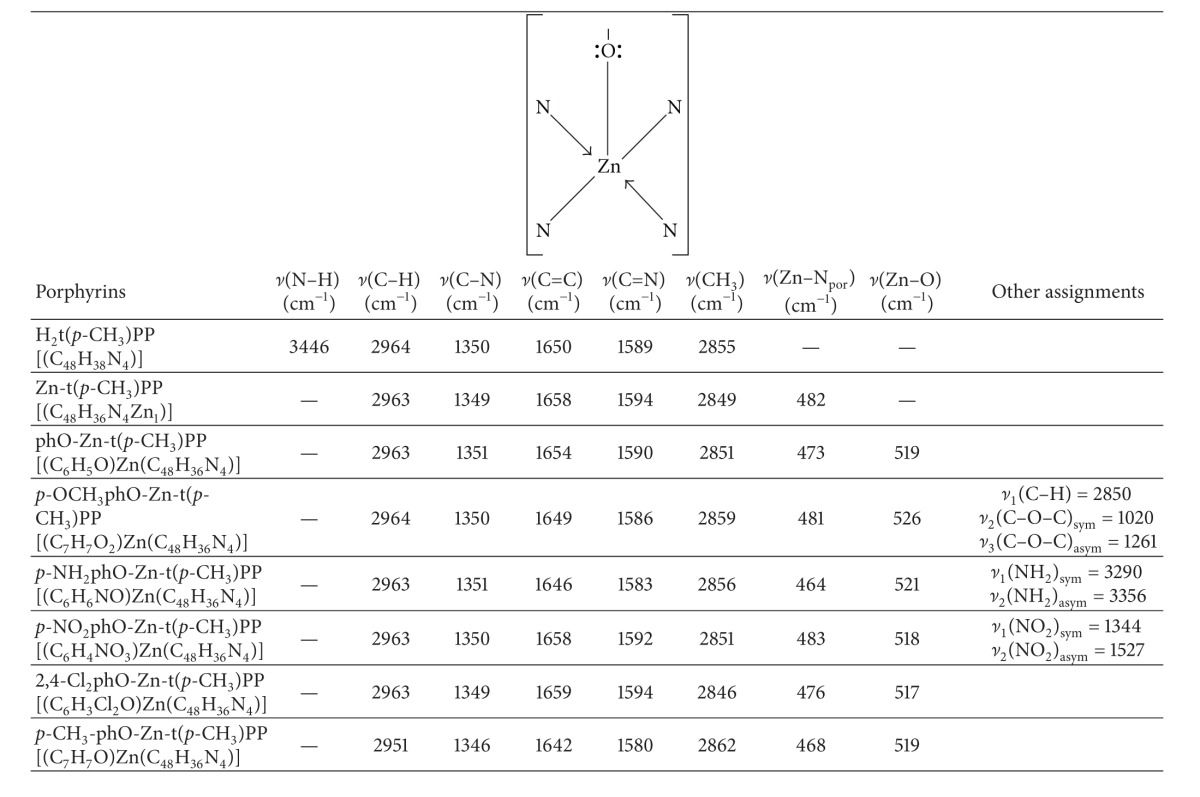

**Table 5:** Mass data (*m/z* ratio) and elemental analytical data of X-Zn-t(*p*-CH_3_)PP (X = different phenols as axial ligand) along with their calculated values.

Porphyrins	Molecular formula	*m/z* ratio calculated (found)	Percentage calculated (found)		
			C	H	N
phO-Zn-t(*p*-CH_3_)PP [(C_6_H_5_O)Zn(C_48_H_36_N_4_)]	C_54_H_41_N_4_ZnO	828.39 (828.29)	78.29 (78.30)	4.99 (4.99)	6.76 (6.76)
*α*-Naphthol-Zn-t(*p*-CH_3_)PP [(C_10_H_7_O)Zn(C_48_H_36_N_4_)]	C_58_H_43_N_4_ZnO	878.90 (878.96)	79.31 (79.17)	4.94 (4.93)	6.38 (6.37)
*p*-NH_2_phO-Zn-t(*p*-CH_3_)PP [(C_6_H_6_NO)Zn(C_48_H_36_N_4_)]	C_54_H_43_N_5_ZnO	843.39 (844.46)	76.81 (76.85)	5.13 (5.14)	8.3 (8.30)
*o*-OCH_3_phO-Zn-t(*p*-CH_3_)PP [(C_7_H_7_O_2_)Zn(C_48_H_36_N_4_)]	C_55_H_43_N_4_ZnO_2_	858.53 (858.49)	76.94 (76.77)	5.05 (5.03)	6.53 (6.51)
*m*-ClphO-Zn-t(*p*-CH_3_)PP [(C_6_H_4_ClO)Zn(C_48_H_36_N_4_)]	C_54_H_40_N_4_ZnOCl	862.95 (862.90)	75.16 (75.08)	4.67 (4.67)	6.49 (6.49)
2,4Cl_2_phO-Zn-t(*p*-CH_3_)PP [(C_6_H_3_Cl_2_O)Zn(C_48_H_36_N_4_)]	C_54_H_39_N_4_ZnOCl_2_	897.39 (897.30)	72.27 (72.36)	4.38 (4.39)	6.24 (6.25)

**Table 6:** Fluorescence spectral data of axially ligated compound of X-Zn-t(*p*-CH_3_)PP (X = different phenols as axial ligands) in methanol solvent using excitation at ~550 nm.

Porphyrins	*λ*_max⁡_ (nm)
Zn-t(*p*-CH_3_)PP [(C_48_H_36_N_4_Zn_1_)]	590, 640
*p*-NH_2_phO-Zn-t(*p*-CH_3_)PP [(C_6_H_6_NO)Zn(C_48_H_36_N_4_)]	612, 660

**Table 7:** *In  vitro* efficacy of axially ligated X-Zn-t(*p*-CH_3_)PP (X = different phenols as axial ligand) against the pathogen *Sclerotium  rolfsi*. Colony diameter of control *C* = 90 mm.

Porphyrins	Different concentration (ppm) of the complexes	Colony diameter (in mm) at different concentrations	% inhibition *I* = [(*C* − *T*)/*C*]∗100* *at different concentrations (ppm)
*p*-NH_2_phO-Zn-t(*p*-CH_3_)PP [(C_6_H_6_NO)Zn(C_48_H_36_N_4_)]	100 200 300	52.62 38.87 28	41.53 56.81 68.88

*p*-CH_3_phO-Zn-t(*p*-CH_3_)PP [(C_7_H_7_O)Zn(C_48_H_36_N_4_)]	100 200 300	49.12 40.25 31.3	45.4 55.27 65.22

*p*-OCH_3_phO-Zn-t(*p*-CH_3_)PP [(C_7_H_7_O_2_)Zn(C_48_H_36_N_4_)]	100 200 300	51 21.87 11.62	43.33 75.69 87.08

*m*-NO_2_phO-Zn-t(*p*-CH_3_)PP [(C_6_H_4_NO_3_)Zn(C_48_H_36_N_4_)]	100 200 300	48 36.5 25.5	46.66 59.44 71.66

*p*-ClphO-Zn-t(*p*-CH_3_)PP [(C_6_H_4_ClO)Zn(C_48_H_36_N_4_)]	100 200 300	37.5 21.12 12.75	58.33 76.52 85

## References

[1] Gupta VK, Chauhan DK, Saini VK, Agarwal S, Antonijevic MM, Lang H (2003). A porphyrin based potentiometric sensor for Zn^2+^ determination. *Sensors*.

[2] Chen C-T (2004). Evolution of red organic light-emitting diodes: materials and devices. *Chemistry of Materials*.

[3] Wolfbeis OS (2006). Fiber-optic chemical sensors and biosensors. *Analytical Chemistry*.

[4] Gupta VK, Chandra S, Chauhan DK, Mangla R (2002). Membranes of 5,10,15,20-tetrakis(4-methoxyphenyl) porphyrinatocobalt (TMOPP-Co) (I) as MoO_4_
^  2−^ -selective sensors. *Sensors*.

[5] Ozoemena KI (2006). Anodic oxidation and amperometric sensing of hydrazine at a glassy carbon electrode modified with cobalt (II) phthalocyanine-cobalt (II) tetraphenylporphyrin (CoPc-(CoTPP)4) supramolecular complex. *Sensors*.

[6] Ikeda O, Yoshinaga K, Lei J (2005). Nitric oxide detection with glassy carbon electrodes coated with charge-different polymer films. *Sensors*.

[7] Purrello R, Raudino A, Monsù Scolaro L, Loisi A, Bellacchio E, Lauceri R (2000). Ternary porphyrin aggregates and their chiral memory. *Journal of Physical Chemistry B*.

[8] Cho HS, Jeong DH, Cho S (2002). Photophysical properties of porphyrin tapes. *Journal of the American Chemical Society*.

[9] Yu J, Wang X, Zhang B, Weng Y, Zhang L (2004). Prolonged excited-state lifetime of porphyrin due to the addition of colloidal SiO_2_ to Triton X-100 micelles. *Langmuir*.

[10] Tangestaninejad S, Habibi MH, Mirkhani V, Moghadam M (2002). Mn (Br_8_TPPS) supported on Amberlite IRA-400 as a robust and efficient catalyst for alkene epoxidation and alkane hydroxylation. *Molecules*.

[11] Girichev EG, Bazanov MI, Mamardashvili NZ, Gjeyzak A (2000). Electrochemical and electrocatalytical properties of 3,7,13,17-tetramethyl- 2,8,12,18-tetrabutylporphyrin in alkaline solution. *Molecules*.

[12] Gao B, Chen Y, Lei Q (2012). Hydroxylationof cyclohexanewith molecular oxygen catalyzed by highly efficient heterogeneous Mn(III) porphyrin catalysts prepared by special synthesis and immobilization method. *Journal of Inclusion Phenomena and Macrocyclic Chemistry*.

[13] O’Connor AE, Gallagher WM, Byrne AT (2009). Porphyrin and nonporphyrin photosensitizers in oncology: preclinical and clinical advances in photodynamic therapy. *Photochemistry and Photobiology*.

[14] Wathier M, Grinstaff MW (2008). Synthesis and properties of supramolecular ionic networks. *Journal of the American Chemical Society*.

[15] Drain CM, Varotto A, Radivojevic I (2009). Self-organized porphyrinic materials. *Chemical Reviews*.

[16] Vlascici D, Cosma EF, Pica EM (2008). Free base porphyrins as ionophores for heavy metal sensors. *Sensors*.

[17] Aviezer D, Cotton S, David M (2000). Porphyrin analogues as novel antagonists of fibroblast growth factor and vascular endothelial growth factor receptor binding that inhibit endothelial cell proliferation, tumor progression, and metastasis. *Cancer Research*.

[18] Imahori H, Mori Y, Matano Y (2003). Nanostructured artificial photosynthesis. *Journal of Photochemistry and Photobiology C*.

[19] Forneli A, Planells M, Sarmentero MA (2008). The role of para-alkyl substituents on meso-phenyl porphyrin sensitised TiO_2_ solar cells: control of the e_TiO2_/electrolyte^+^ recombination reaction. *Journal of Materials Chemistry*.

[20] Adler AD, Longo FR, Finarelli JD, Goldmacher J, Assour J, Korsakoff L (1967). A simplified synthesis for meso-tetraphenylporphin. *Journal of Organic Chemistry*.

[21] Kadish KM, Smith KM, Guilard R (2000). *The Porphyrin Handbook*.

[22] Saito K, Kashiwagi Y, Ohkubo K, Fukuzumi S (2006). An extremely long-lived charge-separated state of zinc tetraphenylporphyrin coordinated with pyridylnaphthalene-diimide. *Journal of Porphyrins and Phthalocyanines*.

[23] Praneeth VKK, Paulat F, Berto TC (2008). Electronic structure of six-coordinate iron(III)-porphyrin NO adducts: the elusive iron(III)-NO(radical) state and its influence on the properties of these complexes. *Journal of the American Chemical Society*.

[24] Semeikin AS, Koifman OI, Berezin BD (1982). Synthesis of tetraphenylporphins with active groups in the phenyl rings. 1. Preparation of tetrakis(4-aminophenyl)porphin. *Chemistry of Heterocyclic Compounds*.

[25] Kadish KM, Smith KM, Guilard R (1999). Biochemistry and binding activation of small molecules. *The Porphyrin Handbook*.

[26] Nappa M, Valentine JS (1978). The influence of axial ligands on metalloporphyrin visible absorption spectra. Complexes of tetraphenylporphinatozinc. *Journal of the American Chemical Society*.

[27] Wang M-YR, Hoffman BM (1984). Systematic trends in metalloporphyrin optical spectra. *Journal of the American Chemical Society*.

[28] Stojiljkovic I, Evavold BD, Kumar V (2001). Antimicrobial properties of porphyrins. *Expert Opinion on Investigational Drugs*.

[29] Rajesh K, Rahiman AK, Bharathi KS, Sreedaran S, Gangadevi V, Narayanan V (2010). Spectroscopic, redox and biological studies of push-pull porphyrins and their metal complees. *Bulletin of the Korean Chemical Society*.

[30] Bozja J, Sherrill J, Michielsen S, Stojiljkovic I (2003). Porphyrin-based, light-activated antimicrobial materials. *Journal of Polymer Science, Part A*.

[31] Sanchez-Delgado RA, Lazardi K, Rincon L, Urbina JA (1993). Toward a novel metal-based chemotherapy against tropical diseases. 1. Enhancement of the efficacy of clotrimazole against Trypanosoma cruzi by complexation to ruthenium in RuCl_2_(clotrimazole)_2_. *Journal of Medicinal Chemistry*.

[32] Tarafder MTH, Jin KT, Crouse KA, Ali AM, Yamin BM, Fun H-K (2002). Coordination chemistry and bioactivity of Ni^2+^, Cu^2+^, Cd^2+^ and Zn^2+^ complexes containing bidentate schiff bases derived from S-benzyldithiocarbazate and the X-ray crystal structure of bis[S-benzyl-*β*-*N*-(5-methyl-2-furylmethylene)dithiocarbazato]cadmium(II). *Polyhedron*.

[33] Sheikh J, Juneja H, Ingle V, Ali P, Hadda TB (2013). Synthesis and in vitro biology of Co(II), Ni(II), Cu(II) and Zinc(II) complexes of functionalized beta-diketone bearing energy buried potential antibacterial and antiviral O,O pharmacophore sites. *Journal of Saudi Chemical Society*.

[34] Platt JR, Klevens HB (1944). Spectroscopy of organic molecules in the vacuum ultraviolet. *Reviews of Modern Physics*.

[35] Gurinovich GP, Sevchenko AN, Solovyev KN Spectroscopy of chlorophyll and allied compounds.

[36] Nafie LA, Pézolet M, Peticolas WL (1973). On the origin of the intensity of the resonant raman bands of differing polarization in heme proteins. *Chemical Physics Letters*.

[37] Vincent JM (1947). Distortion of fungal hyphæ in the presence of certain inhibitors. *Nature*.

[38] Thiantanawat A, Long BJ, Brodie AM (2003). Signaling pathways of apoptosis activated by aromatase inhibitors and antiestrogens. *Cancer Research*.

[39] Tong X, Lin S, Fujii M, Hou D-X (2004). Echinocystic acid induces apoptosis in HL-60 cells through mitochondria-mediated death pathway. *Cancer Letters*.

[40] Scheer H, Katz JJ, Smith KM (1975). Nuclear magnetic resonance spectroscopy of porphyrins and metalloporphyrins. *Porphyrins and Metalloporphyrins*.

[41] Fagadar-Cosma E, Enache C, Armeanu I (2009). The influence of pH over topography and spectroscopic properties of silica hybrid materials embedding meso-tetratolylporphyrin. *Materials Research Bulletin*.

[42] Schweiger K, Goldner M, Huckstadt H, Homborg HZ (1999). Synthese and Eigen Schaften Von *cis* Diacid-ophthalocyaninato(2-)thallaton(III); kristallstruktur von Tetra(n-butyl)ammonium-cisdinitro(O,O′)-and-*cis*-dichlorophthalocyaninato(2-)thallat(III). *Zeitschrift für Anorganische und Allgemeine Chemie*.

[43] Dougherty RC, Waller GR (1972). *Biochemical Applications of Mass Spectrometry*.

[44] Timiriazeff C (1885). Colourless chlorophyll. *Nature*.

[45] Strachan J-P, Gentemann S, Seth J (1997). Effects of orbital ordering on electronic communication in multiporphyrin arrays. *Journal of the American Chemical Society*.

[46] Dharmaraj N, Viswanathamurthi P, Natarajan K (2001). Ruthenium(II) complexes containing bidentate Schiff bases and their antifungal activity. *Transition Metal Chemistry*.

[47] Mishra L, Singh VK (1993). Co(ll), Ni(ll) and Cu(lI) and Zn(lI) complexes with Schiff bases derived from 2-aminobenzimidazoles and pyrazolycarboxaldehyde. *Indian Journal of Chemistry*.

